# Supplementary material for: Mitochondrial hydrogen peroxide positively regulates neuropeptide secretion during diet-induced activation of the oxidative stress response
Source: Nat Commun. 2021 Apr 16;12:2304. doi: 10.1038/s41467-021-22561-x (PMC8052458; doi:10.1038/s41467-021-22561-x)
Supplement: Supplementary file 1 — Supplementary Information [file 41467_2021_22561_MOESM1_ESM.pdf]

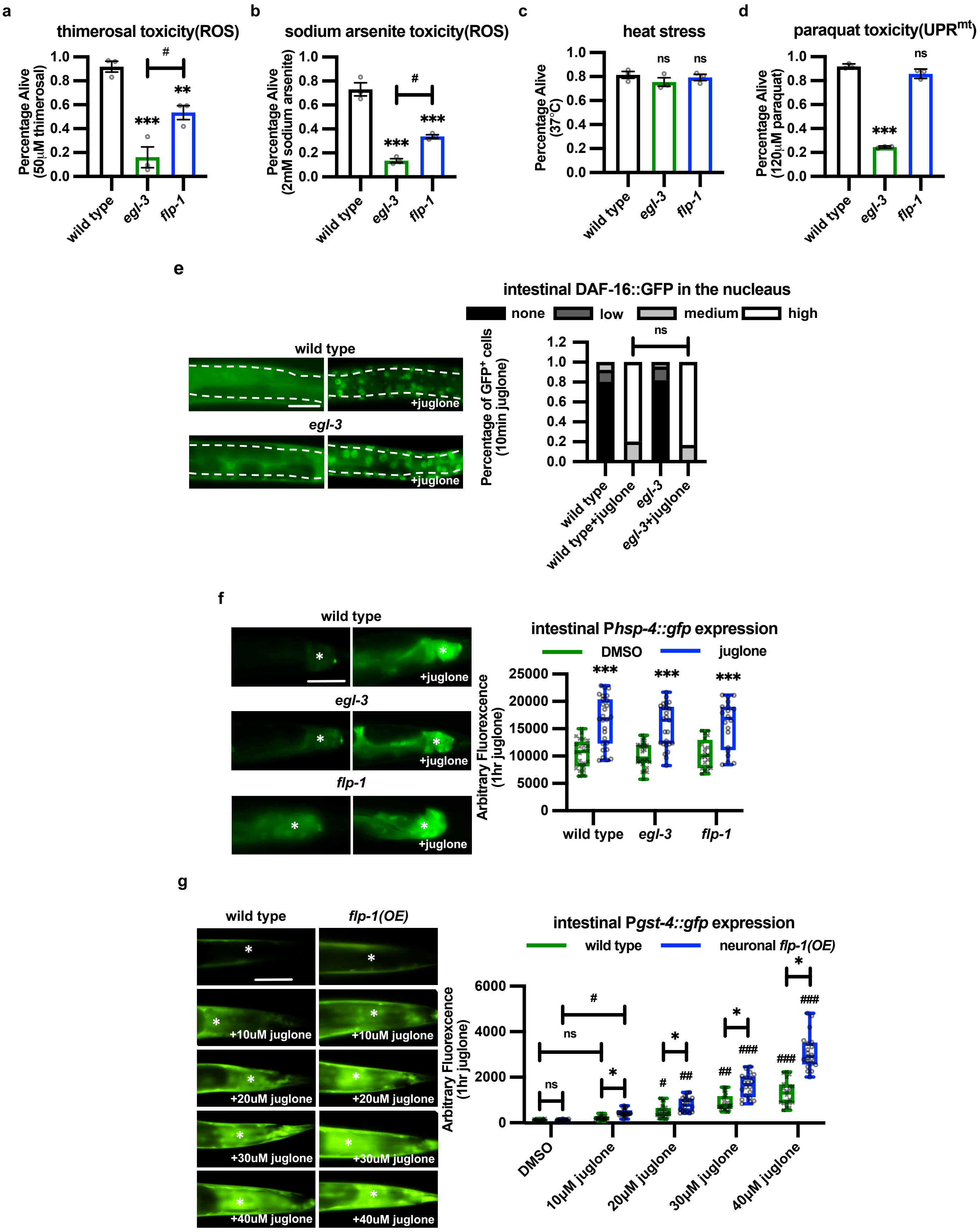
a
thimerosal toxicity(ROS)
Percentage Alive (50μM thimerosal)
wild type
egl-3
flp-1
b
sodium arsenite toxicity(ROS)
Percentage Alive (2mM sodium arsenite)
c
heat stress
Percentage Alive (37°C)
d
paraquat toxicity(UPRmt)
Percentage Alive (120μM paraquat)
e
intestinal DAF-16::GFP in the nucleaus
none
low
medium
high
Percentage of GFP+ cells (10min juglone)
wild type+juglone
egl-3+juglone
+juglone
f
intestinal Phsp-4::gfp expression
DMSO
juglone
Arbitrary Fluorexcence (1hr juglone)
g
intestinal Pgst-4::gfp expression
neuronal flp-1(OE)
flp-1(OE)
+10uM juglone
+20uM juglone
+30uM juglone
+40uM juglone
DMSO
10μM juglone
20μM juglone
30μM juglone
40μM juglone

Supplementary Figure 1. EGL-3 and FLP-1 signaling regulate specific stress responses.

(a-d) Percentage of surviving animals of the indicated strains following treatment of young adults with the indicated stressors and 16 hour recovery. Data are mean values ± s.e.m. n=116, 57, 78; 147, 96, 108; 154, 90, 126; 70, 49, 78 biologically independent samples over 3 independent experiments. *** $P < 0.001$, n.s not significant by Student's two-tailed *t*-test. $^{\#}$ $P < 0.05$ by one-way ANOVA with Dunnett's test. (e) Representative images and quantification of nuclear fluorescence the posterior regions of transgenic animals expressing *daf-16::gfp* after 10min of DMSO or juglone treatment. Fewer than 10, between 11 and 20, and above 20 fluorescent nuclei are denoted Low, Medium, and High, respectively. n=25, 35, 22, 24 biologically independent samples. $^{\#}$ $P < 0.05$ by one-way ANOVA with Dunnett's test. Scale bar: 100μm. (f) Representative images and quantification of the posterior regions of transgenic animals expressing the ER stress marker P*hsp-4::gfp* after 1hour of DMSO or juglone treatment and 4 hours recovery. Asterisks mark the intestinal region used for quantitative analysis. The boxes span the interquartile range, median is marked by the line and whiskers indicate the minimum and the maximum values. n=20 biologically independent samples. *** $P < 0.001$ by Student's two-tailed *t*-test. n.s not significant by one-way ANOVA with Dunett's test. Scale bar: 50μm. (g) Representative images and quantification of P*gst-4::gfp* expression in wild type or *flp-1* overexpressing animals (*flp-1(OE)*) following treatment with the indicated concentrations of juglone or vehicle (DMSO). P*gst-4::gfp* expression in body wall muscles, which appears as fluorescence on the edge of animals in some images, was not quantified. The boxes span the interquartile range, median is marked by the line and whiskers indicate the minimum and the maximum values. n=20, 20, 21, 17, 21, 20, 23, 21, 21, 21 biologically independent samples. * $P < 0.05$, n.s not significant by Student's two-tailed *t*-test. $^{\#}$ $P < 0.05$, $^{\#\#}$ $P < 0.01$, $^{\#\#\#}$ $P < 0.001$ by one-way ANOVA with Dunnett's test. Scale bar: 50μm.

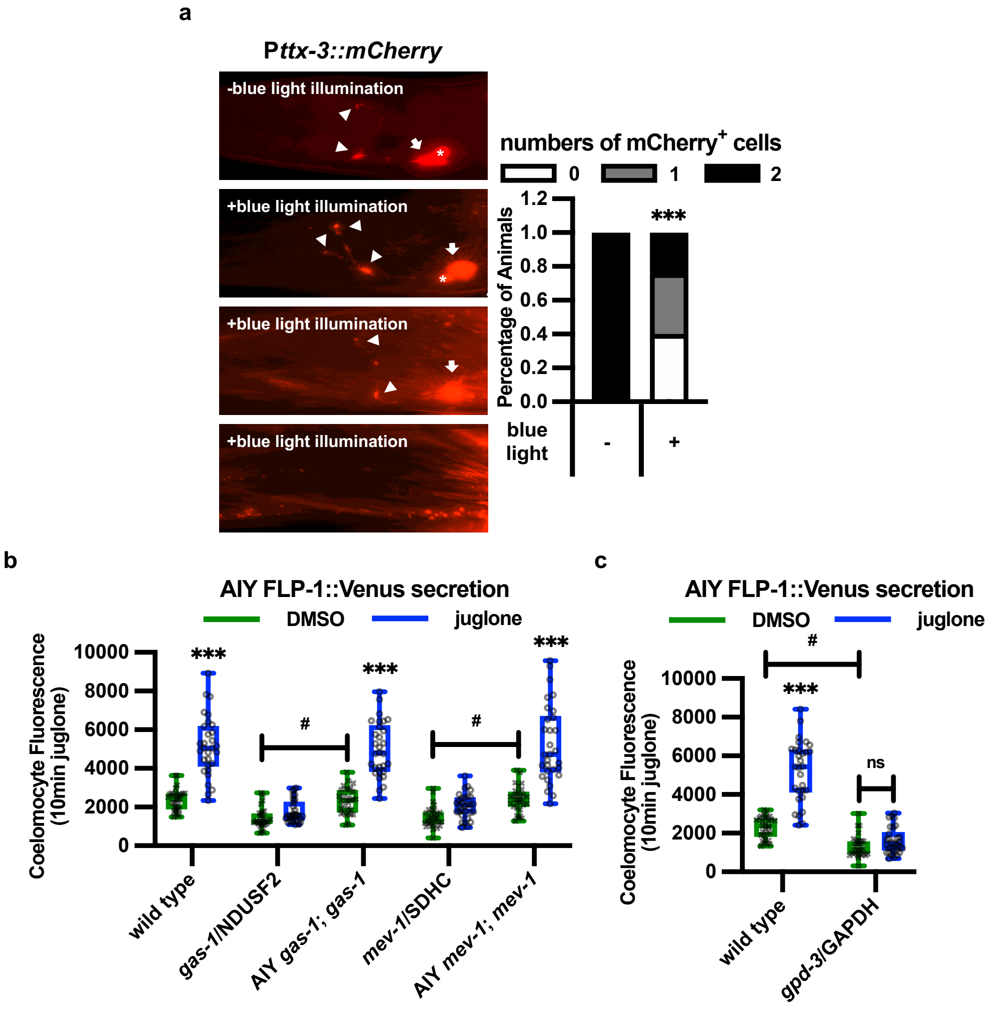

Supplementary Figure 2. mito-miniSOG-induced AIY ablation and effects of ATP-generating mutants on FLP-1 secretion.

(a) Representative images and quantification of mCherry marked AIY neurons from transgenic animals expressing mito-miniSOG under the control *ttx-3* promoter after 30min of 50mW/cm^2 blue light illumination and overnight recovery on NGM plates. Efficiency of mito-miniSOG-induced cell ablation was measured by counting the number of visible mCherry-marked AIY neurons. Arrows indicate somas, asterisks indicate somas of the second AIY and arrowheads indicate axons. n=50 biologically independent samples. *** *P* < 0.001. Scale bar: 10μm. (b) Quantification of average coelomocyte fluorescence of the indicated mutants expressing FLP-1::Venus fusion proteins in AIY following vehicle (DMSO) or juglone treatment for 10 minutes. AIY *gas-1* denotes transgenes expressing *gas-1* under the *ttx-3* promoter. AIY *mev-1* denotes transgenes expressing *mev-1* cDNA under the *ttx-3* promoter. NDUFS2, NADH:ubiquinone oxidoreductase core subunit S2; SDHC, succinate dehydrogenase complex subunit C. The boxes span the interquartile range, median is marked by the line and whiskers indicate the minimum and the maximum values. n=30 biologically independent samples. *** *P* < 0.001 by Student's two-tailed *t*-test. $^{\#}$ *P* < 0.05 by one-way ANOVA with Dunnett's test. (c) Quantification of average

coelomocyte fluorescence of the indicated mutants expressing FLP-1::Venus fusion proteins in AIY following vehicle (DMSO) or juglone treatment for 10 minutes. GAPDH, glyceraldehyde-3-phosphate dehydrogenase. The boxes span the interquartile range, median is marked by the line and whiskers indicate the minimum and the maximum values. n=30 biologically independent samples. *** $P < 0.001$, n.s not significant by Student's two-tailed *t*-test. $^{\#}$ $P < 0.05$ by one-way ANOVA with Dunnett's test.

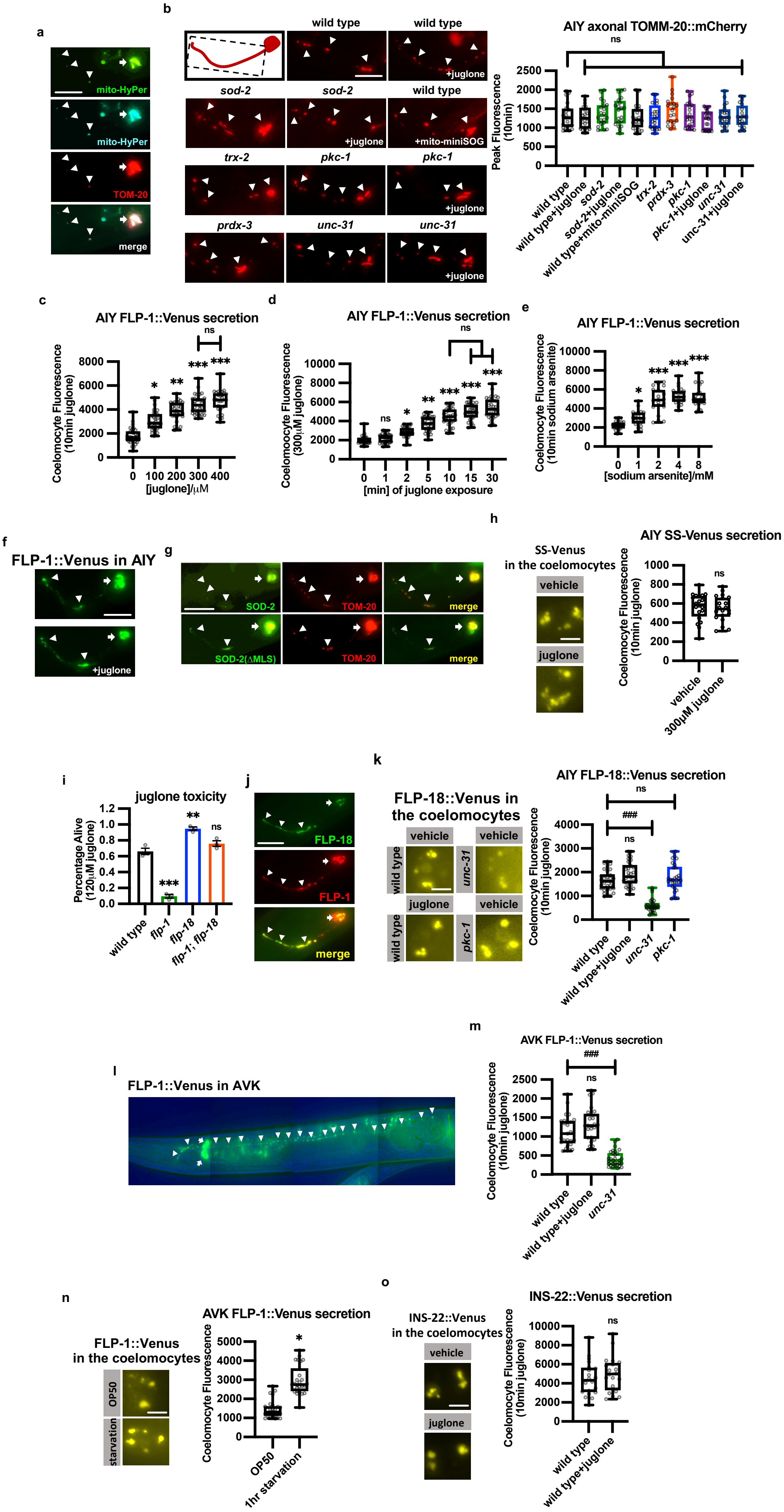
a
mito-HyPer
mito-HyPer
TOM-20
merge
b
wild type
wild type
+juglone
sod-2
sod-2
wild type
+juglone
+mito-miniSOG
trx-2
pkc-1
pkc-1
+juglone
prdx-3
unc-31
unc-31
+juglone
AIY axonal TOMM-20::mCherry
ns
Peak Fluorescence (10min)
wild type
wild type+juglone
sod-2
sod-2+juglone
wild type+mito-miniSOG
trx-2
prdx-3
pkc-1
pkc-1+juglone
unc-31
unc-31+juglone
c
AIY FLP-1::Venus secretion
Coelomocyte Fluorescence (10min juglone)
[juglone]/μM
d
AIY FLP-1::Venus secretion
Coelomocyte Fluorescence (300μM juglone)
[min] of juglone exposure
e
AIY FLP-1::Venus secretion
Coelomocyte Fluorescence (10min sodium arsenite)
[sodium arsenite]/mM
f
FLP-1::Venus in AIY
+juglone
g
SOD-2
TOM-20
merge
SOD-2(ΔMLS)
TOM-20
merge
h
SS-Venus in the coelomocytes
vehicle
juglone
AIY SS-Venus secretion
Coelomocyte Fluorescence (10min juglone)
vehicle
300μM juglone
i
juglone toxicity
Percentage Alive (120μM juglone)
wild type
flp-1
flp-18
flp-1; flp-18
j
FLP-18
FLP-1
merge
k
FLP-18::Venus in the coelomocytes
vehicle
vehicle
wild type
unc-31
juglone
vehicle
wild type
pkc-1
AIY FLP-18::Venus secretion
Coelomocyte Fluorescence (10min juglone)
wild type
wild type+juglone
unc-31
pkc-1
l
FLP-1::Venus in AVK
m
AVK FLP-1::Venus secretion
Coelomocyte Fluorescence (10min juglone)
wild type
wild type+juglone
unc-31
n
FLP-1::Venus in the coelomocytes
OP50
starvation
AVK FLP-1::Venus secretion
Coelomocyte Fluorescence
OP50
1hr starvation
o
INS-22::Venus in the coelomocytes
vehicle
juglone
INS-22::Venus secretion
Coelomocyte Fluorescence (10min juglone)
wild type
wild type+juglone

Supplementary Figure 3. Specificity of juglone-induced FLP-1 secretion from AIY. (a) Representative images showing co-localization of mito-HyPer puncta in the YFP or CFP channels with the mitochondrial marker TOMM-20::mCherry in AIY axons. Scale bar: 10μm. (b) Representative images and quantification of mCherry fluorescence from the indicated mutants co-expressing mito-HyPer and TOMM-20::mCherry in AIY axons with or without 10 minutes of the indicated oxidative stress treatments. The boxes span the interquartile range, median is marked by the line and whiskers indicate the minimum and the maximum values. n=20 biologically independent samples. n.s not significant by one-way ANOVA with Dunnett's test. Scale bar: 10μm. (c) Quantification of average coelomocyte fluorescence intensity following exposure of FLP-1::Venus-expressing animals to the indicated juglone concentrations for 10 minutes. The boxes span the interquartile range, median is marked by the line and whiskers indicate the minimum and the maximum values. n=30 biologically independent samples. * $P < 0.05$, ** $P < 0.01$, *** $P < 0.001$, n.s not significant by Student's two-tailed *t*-test. (d) Quantification of average coelomocyte fluorescence following exposure of FLP-1::Venus-expressing animals to 300uM juglone for the indicated number of minutes. The boxes span the interquartile range, median is marked by the line and whiskers indicate the minimum and the maximum values. n=30 biologically independent samples. * $P < 0.05$, ** $P < 0.01$, *** $P < 0.001$, n.s not significant by Student's two-tailed *t*-test. (e) Quantification of average coelomocyte fluorescence intensity following exposure of FLP-1::Venus-expressing animals to the indicated sodium arsenite concentrations for 10 minutes. The boxes span the interquartile range, median is marked by the line and whiskers indicate the minimum and the maximum values. n=30 biologically independent samples. * $P < 0.05$, *** $P < 0.001$ by Student's two-tailed *t*-test. (f) Representative image of AIY in adult animals expressing FLP-1::Venus in AIY following 10 minutes of vehicle (DMSO) or juglone treatment. Scale bar: 10μm. (g) Representative images showing that SOD-2::GFP puncta co-localized with TOMM-20::mCherry puncta in AIY axons. In contrast, SOD-2(ΔMLS)::GFP fusion proteins adopted a diffuse pattern of localization in AIY axons. Scale bar: 10μm. (h) Representative images and quantification of average coelomocyte fluorescence following exposure of animals expressing constitutively secreted Venus in AIY (P*ttx-3::ss-Venus*) to juglone for 10 minutes. The boxes span the interquartile range, median is marked by the line and whiskers indicate the minimum and the maximum values. n=20 biologically independent samples. n.s not significant by Student's two-tailed *t*-test. Scale bar: 5μm. (i) Percentage of surviving animals of the indicated strains 16 hours following treatment of young adults with juglone for 4 hours. Data are mean values ± s.e.m. n=89, 74, 123, 88 biologically independent samples over 3 independent experiments. ** $P < 0.01$, *** $P < 0.001$, n.s not significant by Student's two-tailed *t*-test. (j) Representative images of AIY axons of adults co-expressing FLP-18::Venus and FLP-1::mCherry. Arrowheads denote axonal puncta and arrows denote the AIY soma. Scale bar: 5μm. (k) Representative images and quantification of average coelomocyte fluorescence following exposure of animals expressing FLP-18::Venus in AIY to juglone. The boxes span the interquartile range, median is marked by the line and whiskers indicate the minimum and the maximum values. n=30 biologically independent samples. $^{###}$ $P < 0.001$, n.s not significant by Student's two-tailed *t*-test. Scale bar: 5μm. (l) Representative image of an adult expressing FLP-1::Venus in AVK using the *flp-1*(513bp) promoter. Arrowheads denote FLP-1::Venus puncta in the AVK axon and arrows denote AVK soma. Scale bar: 30μm.

(m) Quantification of average coelomocyte fluorescence following exposure of animals expressing FLP-1::Venus in AVK to juglone. The boxes span the interquartile range, median is marked by the line and whiskers indicate the minimum and the maximum values. n=25, 25, 30 biologically independent samples. $^{###}$ $P < 0.001$, n.s not significant by Student's two-tailed *t*-test. (n) Representative images and quantification of average coelomocyte fluorescence following exposure of animals expressing FLP-1::Venus in AVK to starvation for 1 hour. The boxes span the interquartile range, median is marked by the line and whiskers indicate the minimum and the maximum values. n=25 biologically independent samples. * $P < 0.05$ by Student's two-tailed *t*-test. Scale bar: 5μm. (o) Representative images and quantification of average coelomocyte fluorescence following exposure of animals expressing INS-22::Venus in motor neurons (under the *unc-129* promoter) to juglone. The boxes span the interquartile range, median is marked by the line and whiskers indicate the minimum and the maximum values. n=20 biologically independent samples. n.s not significant by Student's two-tailed *t*-test. n.s not significant by Student's two-tailed *t*-test. Scale bar: 5μm.

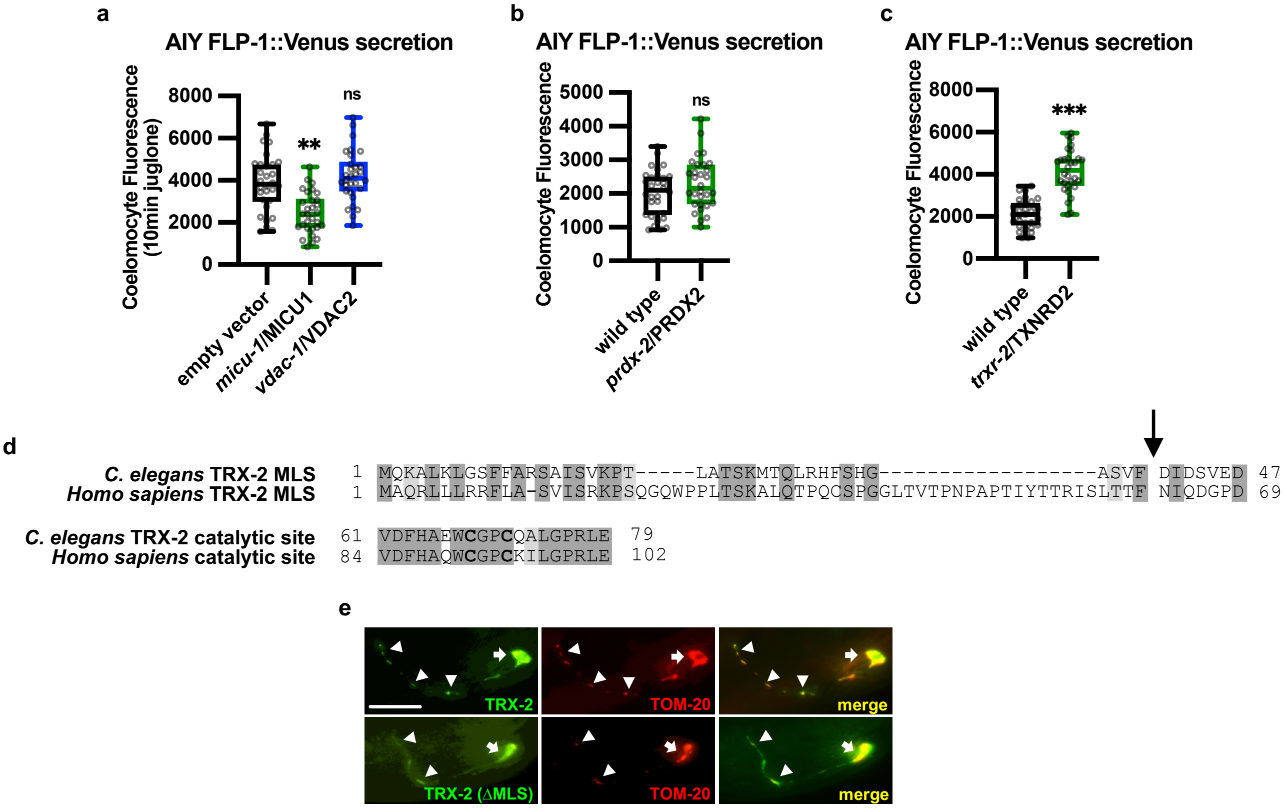

Supplementary Figure 4. The peroxiredoxin-thioredoxin system negatively regulates FLP-1::Venus secretion from AIY.

(a) Quantification of average coelomocyte fluorescence intensity in animals subject to RNAi-mediated knockdown of the indicated genes. The boxes span the interquartile range, median is marked by the line and whiskers indicate the minimum and the maximum values. n=20 biologically independent samples. ** *P* < 0.01, n.s not significant by Student's two-tailed *t*-test. (b) Quantification of average coelomocyte fluorescence intensity of *prdx-2*/peroxiredoxin2 mutants expressing FLP-1::Venus in AIY. The boxes span the interquartile range, median is marked by the line and whiskers indicate the minimum and the maximum values. n=30 biologically independent samples. n.s not significant by Student's two-tailed *t*-test. (c) Quantification of average coelomocyte fluorescence intensity of *trxr-2*/thioredoxin reductase2 mutants expressing FLP-1::Venus in AIY. The boxes span the interquartile range, median is marked by the line and whiskers indicate the minimum and the maximum values. n=30 biologically independent samples. *** *P* < 0.001, n.s not significant by Student's two-tailed *t*-test. (d) Amino acid sequence alignment of human TRX2 and *C. elegans* TRX-2 showing the mitochondrial localization sequence that was truncated (arrow) and the positions of the conserved cysteines (bold) that were mutated to alanine to generate catalytically inactive TRX-2 (C68A, C71A). (e) Representative images of AIY in animals co-expressing the mitochondrial marker TOMM-20::mCherry and either wild type TRX-2::GFP or TRX-2(ΔMLS)::GFP, which lacks the TRX-2 mitochondrial localization sequence. Scale bar: 10μm.

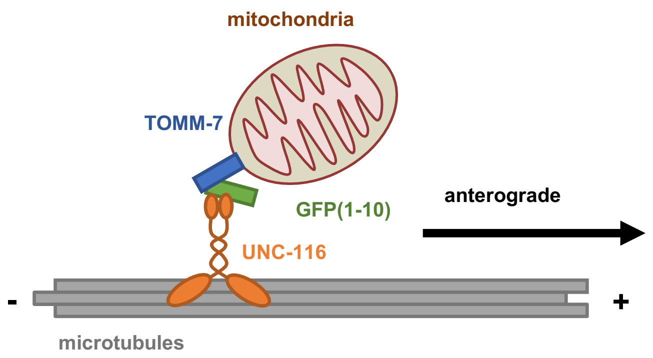

Supplementary Figure 5. TOMM-7::UNC-116 fusion protein schematic
Schematic showing how TOMM-7::UNC-116 fusion proteins can restore anterograde mitochondrial trafficking to ric-7 mutants, adapted from (RAWSON *et al.* 2014).

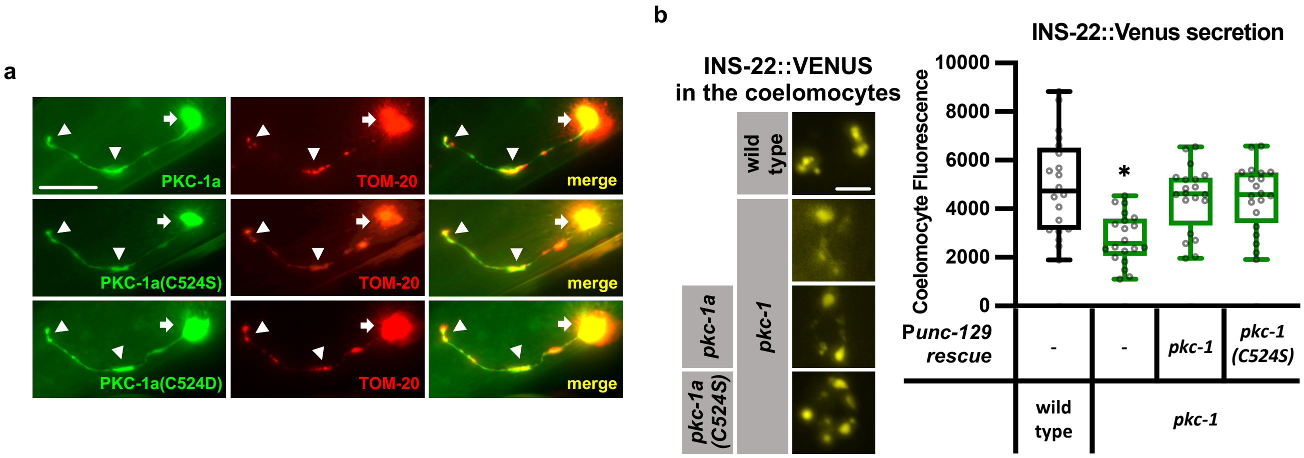

Spplementary Figure 6. Localization of PKC-1 in AIY and juglone-induced INS-22 secretion.

(a) Representative images of AIY axons from animals co-expressing the indicated PKC-1::GFP fusion proteins and TOMM-20::mCherry under the *ttx-3* promoter. Scale bar: 10μm. (b) Representative images and quantification of average coelomocyte fluorescence intensity of the indicated mutants expressing INS-22::Venus in DA/DB motor neurons (under the *unc-129* promoter) following 10 minute treatment with juglone. The boxes span the interquartile range, median is marked by the line and whiskers indicate the minimum and the maximum values. n=20 biologically independent samples. * $P < 0.05$ by Student's two-tailed *t*-test. n.s not significant by one-way ANOVA with Dunnett's test. Scale bar: 5μm.

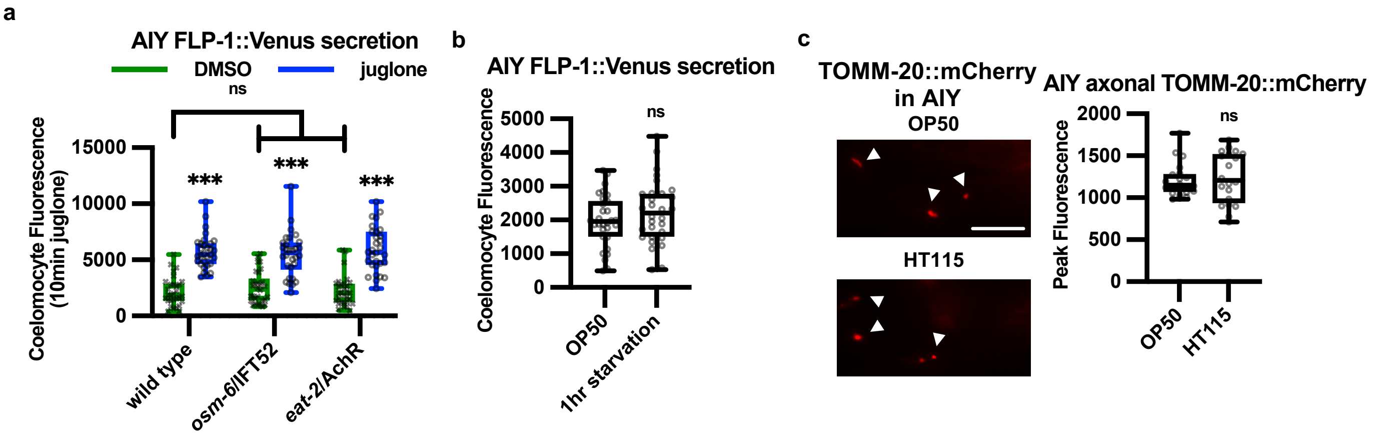

Supplementary Figure 7. Sensory input or starvation do not impact FLP-1 secretion.

(a) Quantification of average coelomocyte fluorescence intensity of the indicated mutants expressing FLP-1::Venus in AIY following juglone treatment. The boxes span the interquartile range, median is marked by the line and whiskers indicate the minimum and the maximum values. n=30 biologically independent samples. *** $P < 0.001$ by Student's two-tailed *t*-test. n.s not significant by one-way ANOVA with Dunnett's test. (b) Quantification of average coelomocyte fluorescence intensity of the indicated mutants expressing FLP-1::Venus in AIY following 1 hour starvation. The boxes span the interquartile range, median is marked by the line and whiskers indicate the minimum and the maximum values. n=30 biologically independent samples. n.s not significant by Student's two-tailed *t*-test. (c) Representative images and quantification of TOMM-20:mCherry punctal fluorescence in AIY axons of animals grown on OP50 or HT115 bacteria. The boxes span the interquartile range, median is marked by the line and whiskers indicate the minimum and the maximum values. n=20 biologically independent samples. n.s not significant by Student's two-tailed *t*-test. Scale bar: 10μm.

Table S1: neuropeptide screening for juglone toxicity

RNAi	Juglone Survival**	RNAi	Juglone Survival**
egl-3	20%*	nlp-14	91%
flp-1	63%*	nlp-15	88%
flp-2	72%*	nlp-16	81%
flp-3	85%	nlp-17	86%
flp-4	96%	nlp-18	96%
flp-5	102%	nlp-19	91%
flp-6	124%*	nlp-20	90%
flp-7	83%	nlp-21	122%*
flp-8	103%	nlp-22	99%
flp-9	93%	nlp-23	101%
flp-10	94%	nlp-24	93%
flp-11	85%	nlp-25	84%
flp-12	72%*	nlp-26	105%
flp-13	89%	nlp-27	96%
flp-14	89%	nlp-28	96%
flp-15	94%	nlp-29	99%
flp-16	88%	nlp-30	99%
flp-17	98%	nlp-31	95%
flp-18	109%	nlp-32	98%
flp-19	101%	nlp-33	97%
flp-20	95%	nlp-34	100%
flp-21	102%	nlp-35	102%
flp-22	78%	nlp-36	104%
flp-23	102%	nlp-37	83%
flp-24	86%	nlp-38	95%
flp-25	113%	nlp-39	96%
flp-26	101%	nlp-40	90%
flp-27	100%	nlp-41	93%
flp-28	98%	nlp-42	104%
flp-32	83%	nlp-43	95%
flp-33	98%	nlp-44	92%
flp-34	90%	nlp-45	108%
nlp-1	94%	nlp-46	100%
nlp-2	89%	nlp-47	101%
nlp-3	67%*	nlp-48	124%*
nlp-4	108%	nlp-49	97%
nlp-5	103%	nlp-50	97%

**nlp-6**	95%	**nlp-51**	92%
**nlp-7**	95%	**nlp-52**	94%
**nlp-8**	99%	**nlp-53**	98%
**nlp-9**	88%	**nlp-54**	114%
**nlp-10**	76%*	**nlp-55**	60%*
**nlp-11**	91%	**nlp-56**	109%
**nlp-12**	93%	**nlp-57**	103%
**nlp-13**	99%		

*****denotes $p < 0.05$, compared to empty vector controls

******denotes percentage of surviving animals normalized to empty vector controls

animals were fed with bacteria expressing dsRNA corresponding to the indicated genes

Table S2: GPCR screening for juglone toxicity

RNAi	Juglone Survival**	mutants	Juglone Survival**	reference
**GPCR expressed in the intestine**				
**npr-20**	96%			(Cao et al., 2017)
**npr-28**	104%			(Cao et al., 2017)
**nmur-4**	84%	ok1381	98%	(Cao et al., 2017)
**npr-23**	95%			(Cao et al., 2017; Kaletsky et al., 2018)
**npr-26**	101%			(Cao et al., 2017)
**npr-8**	94%			(Cao et al., 2017)
**npr-4**	68%*	tm1782	25%	(Cao et al., 2017; Kaletsky et al., 2018)
**npr-12**	94%			(Cao et al., 2017)
**frpr-19**	91%			(Cao et al., 2017)
**npr-30**	92%			(Cao et al., 2017)
**W10C4.1**	59%*			(Kaletsky et al., 2018)
**gnrr-2**	64%*			(Kaletsky et al., 2018)
**M04G7.3**	107%			(Kaletsky et al., 2018)
**lat-1**	101%			(Kaletsky et al., 2018)
**pdfr-1**	99%			(Kaletsky et al., 2018)
**lat-2**	104%			(Kaletsky et al., 2018)
**B0334.6**	99%			(Kaletsky et al., 2018)
**F32D8.10**	70%*			(Kaletsky et al., 2018)
**dmsr-1**	148%*			(Kaletsky et al., 2018)
**known FLP-1 GPCRs**				
**frpr-7**	84%	vj290	102%	(Oranth et al., 2018)
**npr-6**	74%*	vj288	102%	(Oranth et al., 2018)
**GPCR that function in the intestine for other pathways**				
**npr-22**	82%	ok1598	98%	(Palamiuc et al., 2017)
**npr-5**	89%	ok1583	89%	(Bhardwaj et al., 2020)

*denotes $p < 0.05$, compared to empty vector controls

**denotes percentage of surviving animals normalized to empty vector animals were fed with bacteria expressing dsRNA corresponding to the

Supplementary Table 3. Reagents

**Bacterial and Virus Strains**		
OP50 *Escherichia coli*	Caenorhabditis Genetics Center	WBStrain00041969
HT115(DE3) *Escherichia coli*	Caenorhabditis Genetics Center	WBStrain00041079
**Chemicals, Peptides, and Recombinant Proteins**		
juglone(5-Hydroxy-1,4-naphthlenedione)	Calbiochem	CAS 481-39-0<br>Cat# 420120-250MG
thimerosal(2-(Ethylmercuriomercapto)benzoic acid sodium salt)	Millipore Sigma	CAS 54-64-8<br>Cat# 71230-50G
Sodium Arsenite, 0.5% (w/v) Aqueous Solution	Thermo Fisher Scientific	CAS 7784-46-5<br>Cat# 7140-16
paraquat(1,1′-Dimethyl-4,4′-bipyridinium dichloride)	Millipore Sigma	CAS 75365-73-0<br>Cat# 856177-250MG
**Experimental Models: Organisms/Strains**		
EU1 *skn-1(zu67)/nT1*	Caenorhabditis Genetics Center	WBStrain00007249
KP3905 *unc-17(e113)*	this paper	N/A
OJ1218 *unc-25(e156)*	this paper	N/A
MT6308 *eat-4(ky5)*	Caenorhabditis Genetics Center	WBStrain00027259
GR1321 *tph-1(mg280)*	Caenorhabditis Genetics Center	WBStrain00007901
CB1112 *cat-2(e1112)*	Caenorhabditis Genetics Center	WBStrain00004246
MT13113 *tdc-1(n3419)*	Caenorhabditis Genetics Center	WBStrain00027424
MT9455 *tbh-1(n3247)*	Caenorhabditis Genetics Center	WBStrain00027363
OJ2424 *egl-3(nr3090)*	this paper	N/A
OJ2031 *egl-21(n476)*	this paper	N/A
KP3387 *sbt-1(ok901)*	this paper	N/A
KP2753 *pkc-1(nj3)*	this paper	N/A
OJ6555 *flp-1(ok2811)*	this paper	N/A
KP3948 *eri-1(mg366)*; *lin-15b(n744)*	Caenorhabditis Genetics Center	WBStrain00023637
OJ2439 *npr-4(tm1782)*	this paper	N/A
OJ2497 *flp-1(ok2811)*; *npr-4(tm1782)*	this paper	N/A
AX1410 flp-18(db99)	Caenorhabditis Genetics Center	WBStrain00000308
OJ2210 *flp-1(ok2811)*; *flp-18(db99)*	this paper	N/A
KP3948 *eri-1(mg366)*; *lin-15b(n744)*	Caenorhabditis Genetics Center	WBStrain00023637
OJ1531 *idIs7*[*skn-1B/C*::GFP;*rol-6(su1006)*]; *glo-1(zu391)*	this paper	N/A
OJ2176 *egl-3*; *idIs7*; *glo-1*	this paper	N/A
OJ2280 *flp-1*; *idIs7*; *glo-1*	this paper	N/A
OJ2515 *vjEx958*[P*flp-1*::*flp-1(genomic)*]; *flp-1*; *idIs7*; *glo-1*	this paper	N/A

OJ2608 *vjEx970*[P*flp-1*::*egl-3C*::GFP]; *egl-3*	this paper	N/A
OJ2173 *vjEx823*[P*ges-1*::*egl-3C*::GFP]; *egl-3*	this paper	N/A
OJ2864 *vjEx958*; *flp-1*	this paper	N/A
CL2166 *dvIs19*[pAF15(P*gst-4*::GFP::NLS)]	Caenorhabditis Genetics Center	WBStrain00005102
OJ2536 *egl-3*; *dvIs19*	this paper	N/A
OJ2538 *vjEx970*; *egl-3*; *dvIs19*	this paper	N/A
OJ2547 *flp-1*; *dvIs19*	this paper	N/A
OJ2544 *vjEx958*; *flp-1*; *dvIs19*	this paper	N/A
OJ2609 *vjEx972*[P*flp-1*::*egl-3C*::GFP]; *egl-3*; *flp-1*; *dvIs19*	this paper	N/A
OJ2490 *vjEx979*[P*flp-1*::*flp-1B*]; *flp-1*	this paper	N/A
OJ2829 *vjIs120*[P*flp-1*::*flp-1(genomic)*::Venus]	this paper	N/A
OJ2493 *vjEx982*[P*flp-1*::*flp-1(P1+P2+P3+P4)*]; *flp-1*	this paper	N/A
OJ2688 *vjEx1039*[P*flp-1*::*flp-1(P1+P2+P3)*]; *flp-1*	this paper	N/A
OJ2694 *vjEx1045*[P*flp-1*::*flp-1(P1+P2)*]; *flp-1*	this paper	N/A
OJ2700 *vjEx1051*[P*flp-1*::*flp-1(P1)*]; *flp-1*	this paper	N/A
OJ2704 *vjEx1055*[P*flp-1*::*flp-1(SS)*]; *flp-1*	this paper	N/A
TJ356 *zIs356*[*daf-16A/B*::GFP;*rol-6(su1006)*]	Caenorhabditis Genetics Center	WBStrain00034892
OJ2344 *egl-3*; *zIs356*	this paper	N/A
SJ4100 *zcIs13*[*hsp-6*::GFP]	Caenorhabditis Genetics Center	WBStrain00034068
OJ4398 *egl-3*; *zcIs13*	this paper	N/A
OJ2542 *vjIs120*; *dvIs19*	this paper	N/A
OJ2638 *vjEx1016*[P*flp-1*::mito-miniSOG; P*ttx-3*::RFP]	this paper	N/A
OJ2641 *vjEx1019*[P*flp-1(513)*::mito-miniSOG]	this paper	N/A
OJ2644 *vjEx1022*[P*flp-18*::mito-miniSOG; P*ttx-3*::RFP]	this paper	N/A
CZ14478 *juEx1337*[P*nmr-1*::mito-miniSOG; P*nmr-1*::mCherry]	(Yingchuan B. Qi et al., 2012)	N/A
CZ15166 *juEx3802*[P*sra-11*::mito-miniSOG; P*sra-11*::mCherry]	(Yingchuan B. Qi et al., 2012)	N/A
OJ2852 *vjEx1081*[P*ttx-3*::mito-miniSOG; P*ttx-3*::RFP]	this paper	N/A
OJ7277 *vjEx1146*[P*ttx-3*::TeTx]	this paper	N/A
OJ7278 *vjEx1091*[P*ttx-3*::*egl-3C*::GFP]; *egl-3*	this paper	N/A
OJ7279 *vjEx1091*; *egl-3*; *flp-1*	this paper	N/A
OJ7280 *vjIs120*; *flp-1*	this paper	N/A
OJ3614 *vjIs150*[P*ttx-3*::*flp-1(genomic)*::Venus; P*ofm-1*::mCherry]	this paper	N/A
OJ3615 *vjIS151*[P*ttx-3*::*flp-1(genomic)*::Venus; P*ofm-1*::mCherry]	this paper	N/A
OJ7281 *vjEx1124*[P*ttx-3*::ChR2::GFP]; *vjIs150*	this paper	N/A
OJ3809 *unc-31(e928)*; *vjIs150*	this paper	N/A
OJ4511 *unc-2(lj1)*; *vjIs150*	this paper	N/A
OJ7282 *npr-4*; *vjIs150*	this paper	N/A
OJ2676 *vjEx1032*[P*ofm-1*::*npr-4A*]; *npr-4*	this paper	N/A
OJ2678 *vjEx1034*[P*rab-3*::*npr-4A*]; *npr-4*	this paper	N/A

OJ2859 *vjEx1088*[P*ges-1*::*npr-4A*]; *npr-4*	this paper	N/A
OJ2537 *npr-4*; *dvIs19*	this paper	N/A
OJ2549 *flp-1*; *npr-4*; *dvIs19*	this paper	N/A
OJ7283 *npr-4*; *vjIs120*; *dvIs19*	this paper	N/A
OJ4737 *vjIs217*[P*ttx-3*::mito-HyPer; P*ttx-3*::*tomm-20*::mCherry]	this paper	N/A
OJ4737 *vjIs218*[P*ttx-3*::mito-HyPer; P*ttx-3*::*tomm-20*::mCherry]	this paper	N/A
OJ5238 *sod-2(ok1030)*; *vjIs217*	this paper	N/A
OJ7284 *vjEx1081*; *vjIs218*	this paper	N/A
OJ4739 *trx-2(tm2720)*; *vjIs218*	this paper	N/A
OJ5248 *pkc-1*; *vjIs217*	this paper	N/A
OJ5236 *unc-31*; *vjIs217*	this paper	N/A
OJ5304 *vjIs287*[P*ttx-3*::*flp-18*::Venus; P*ofm-1*::mCherry]	this paper	N/A
OJ7285 *unc-31*; *vjIs287*	this paper	N/A
OJ7286 *vjEx1138*[P*ttx-3*::ss-Venus]	this paper	N/A
OJ5730 *vjEx1700*[P*ttx-3*::*flp-1(genomic)*::mCherry; P*ttx-3*::*flp-18(genomic)*::Venus]	this paper	N/A
OJ6376 *vjEx2015*[P*flp-1(513)*::*flp-1(genomic)*::Venus; P*ofm-1*::mCherry]	this paper	N/A
OJ6633 *unc-31*; *vjEx2015*	this paper	N/A
OJ221 *nuIs195*[P*unc-129*::*ins-22*::Venus]	this paper; (Sieburth et al., 2006)	N/A
OJ4515 *sod-2*; *vjIs150*	this paper	N/A
OJ4235 *vjEx1391*[P*ttx-3*::*sod-2*]; *sod-2*; *vjIs150*	this paper	N/A
OJ5665 *vjEx1664*[P*ttx-3*::*sod-2(ΔMLS)*]; *sod-2*; *vjIs150*	this paper	N/A
OJ3806 *ric-7(n2657)*; *vjIs150*	this paper	N/A
OJ7287 *vjEx1081*; *vjIs150*	this paper	N/A
OJ7288 *vjEx1081*; *sod-2*; *vjIs150*	this paper	N/A
OJ7289 *vjEx1081*; *ric-7*; *vjIs150*	this paper	N/A
OJ7290 *vjEx1081*; *unc-31*; *vjIs150*	this paper	N/A
OJ7291 *prdx-3(gk529)*; *vjIs150*	this paper	N/A
OJ4506 *trx-2*; *vjIs150*	this paper	N/A
OJ4201 *vjEx1361*[P*ttx-3*::*trx-2A*]; *trx-2*; *vjIs150*	this paper	N/A
OJ4209 *vjEx1369*[P*ttx-3*::*trx-2A(ΔMLS)*]; *trx-2*; *vjIs150*	this paper	N/A
OJ4212 *vjEx1372*[P*ttx-3*::*trx-2A(ΔCAT)*]; *trx-2*; *vjIs150*	this paper	N/A
OJ7292 *sod-1(tm783)*; *vjIs150*	this paper	N/A
OJ4512 *sod-3(tm760)*; *vjIs150*	this paper	N/A
OJ7293 *sod-4(gk101)*; *vjIs150*	this paper	N/A
OJ7294 *sod-5(tm1146)*; *vjIs150*	this paper	N/A
OJ4805 *vjEx1503*[P*ttx-3*::*sod-2*::GFP; P*ttx-3*::*tomm-20*::mCherry]	this paper	N/A
OJ5734 *vjEx1704*[P*ttx-3*::*sod-2(ΔMLS)*::GFP; P*ttx-3*::*tomm-20*::mCherry]	this paper	N/A
OJ7295 *prdx-2(gk169)*; *vjIs150*	this paper	N/A
OJ4215 *vjEx1375*[P*ttx-3*::*trx-2A*::GFP; P*ttx-3*::*tomm-20*::mCherry]	this paper	N/A

OJ4259 *vjEx1416*[P*ttx-3*::*trx-2A(ΔMLS)*::GFP; P*ttx-3*::*tomm-20*::mCherry]	this paper	N/A
OJ4014 *trxr-2(tm2407)*; *vjIs151*	this paper	N/A
OJ4734 *vjIs213*[P*ttx-3*::*flp-1(genomic)*::Venus; P*ttx-3*::*tomm-20*::mCherry]	this paper	N/A
OJ4733 *vjIs212*[P*ttx-3*::*flp-1(genomic)*::Venus; P*ttx-3*::*tomm-20*::mCherry]	this paper	N/A
OJ5274 *ric-7*; *vjIs213*	this paper	N/A
OJ5250 *unc-104(e1265)*; *vjIs212*	this paper	N/A
OJ5787 *ric-7*; *unc-104*; *vjIs213*	this paper	N/A
OJ5739 *vjEx1709*[P*ttx-3*::mito-truck]; *ric-7*; *vjIs213*	this paper	N/A
OJ7296 *unc-104*; *vjIs150*	this paper	N/A
OJ5786 *ric-7*; *unc104*; *vjIs150*	this paper	N/A
OJ5736 *vjEx1706*[P*ttx-3*::mito-truck]; *ric-7*; *vjIs150*	this paper	N/A
OJ3827 *pkc-1*; *vjIs150*	this paper	N/A
OJ4510 *trx-2*; *pkc-1*; *vjIs150*	this paper	N/A
OJ4223 *vjEx1383*[P*ttx-3*::*pkc-1A*]; *pkc-1*; *vjIs150*	this paper	N/A
OJ4233 *vjEx1389*[P*ttx-3*::*pkc-1A(A160E)*]; *vjIs150*	this paper	N/A
OJ5725 *vjEx1389*; *ric-7*; *vjIs150*	this paper	N/A
OJ5726 *vjEx1389*; *sod-2*; *vjIs150*	this paper	N/A
OJ5727 *vjEx1389*; *unc-104*; *vjIs150*	this paper	N/A
OJ4818 *vjEx1516*[P*ttx-3*::*pkc-1A(C524S)*]; *pkc-1*; *vjIs150*	this paper	N/A
OJ5678 *vjEx1677*[P*ttx-3*::*pkc-1A(C524D)*]; *pkc-1*; *vjIs150*	this paper	N/A
KP4103 *pkc-1*; *nuIs195*	this paper; (Sieburth et al., 2006)	N/A
OJ7297 *vjEx2569*[P*unc-129::pkc-1A*]; *pkc-1*; *vjIs150*	this paper	N/A
OJ5742 *vjEx1712*[P*unc-129*::*pkc-1A(C524S)*]; *pkc-1*; *vjIs150*	this paper	N/A
OJ5750 *vjEx1720*[P*ttx-3*::GFP-linker::*pkc-1A*; P*ttx-3*::*tomm-20*::mCherry]	this paper	N/A
OJ6371 *vjEx2009*[P*ttx-3*::GFP-linker::*pkc-1A(C524S)*; P*ttx-3*::*tomm-20*::mCherry]	this paper	N/A
OJ6374 *vjEx2012*[P*ttx-3*::GFP-linker::*pkc-1A(C524D)*; P*ttx-3*::*tomm-20*::mCherry]	this paper	N/A
OJ4012 *osm-6(p811)*; *vjIs150*	this paper	N/A
OJ4010 *eat-2(ad1113)*; *vjIs150*	this paper	N/A
**Oligonucleotides**		
forward primer for *flp-1* promoter ccccccGCATGCTATAGTTCCATCAACACATCC	this paper	N/A
reverse primer for *flp-1* promoter ccccccGATAAAGTGAAGAAAACCAA	this paper	N/A
forward primer for *flp-1* cDNA/genomic DNA ccccccCCTAGGaaaaATGACTCTGCTCTACCAAG	this paper	N/A
reverse primer for *flp-1* cDNA/genomic DNA ccccccACTAGTTGCGACCAAATTTACTATGGG	this paper	N/A

reverse primer for *flp-1B(P1+P2+P3)* ccccccACCGGTCTTTCCGAATCGCAAGAAG	this paper	N/A
reverse primer for *flp-1B(P1+P2)* ccccccACCGGTTCGTCCGATCGAAGGA	this paper	N/A
reverse primer for *flp-1B(P1)* ccccccACCGGTTCGCCCATATCTCATAAAGT	this paper	N/A
reverse primer for *flp-1B(SS)* ccccccACCGGTTCTCTTTTCCATCTTTTGCAG	this paper	N/A
forward primer for mito-miniSOG ccccccGCTAGCaaaaATGTCGGACACAATTCTTGG	this paper	N/A
reverse primer for mito-miniSOG ccccccGGTACCTTATCCGGAAGATCCTCCAT	this paper	N/A
forward primer for *flp-1* promoter ccccccTCCAACTATAGTTCCATCAACACATCC	this paper	N/A
reverse primer for *flp-1* promoter ccccccGGGCCCTGAAGAAAACCAA	this paper	N/A
reverse primer for *flp-1(513)* promoter ccccccGGATCCGATAAAGTGAAGAAAACCAATGAAG	this paper	N/A
forward primer for *flp-18* promoter ccccccGCATGCAATCGGAGGTAGGTTTGAAAAA	this paper	N/A
reverse primer for *flp-18* proter ccccccGGATCCGTCTAACCCTGAAATTATTATTTTTA G	this paper	N/A
forward primer for *npr-4A* cDNA ccccccGCTAGCaaaaATGTTACTGGAAATTGGCAC	this paper	N/A
reverse primer for *npr-4A* cDNA ccccccGGTACCTTAGAAAGAAGCCTTCCTTGGTAGC	this paper	N/A
forward primer for mito-HyPer ccccccGCTAGCAAAAATGTCCGTCCTGACGCCGC	this paper	N/A
reverse primer for mito-HyPer ccccccCCATGGTTAAACCGCCTGTTTTAAAACTTTAT CGAAATGGC	this paper	N/A
forward primer for *flp-18* genomic DNA ccccccGCTAGCaaaaATGCAACGGTGGTCGGGCGT	this paper	N/A
reverse primer for *flp-18* genomic DNA cccccACCGGTGTTCTCCGATTCGGACGGAAG	this paper	N/A
forward primer for *trx-2A* cDNA ccccccGCTAGCaaaaATGCAGAAAGCACTTAAGCT	this paper	N/A
reverse primer for *trx-2A* cDNA ccccccGGTACCTTAAGCAGCGAGAACGTCCT	this paper	N/A
forward primer for *trx-2A(ΔMLS)* ccccccGCTAGCaaaaATGGACATTGATTCTGTTGAAG AT	this paper	N/A
forward primer for *trx-2A(ΔCAT)* TTGATTTCCACGCAGAATGGTcaGGACCGTcaCAGG CTTTGG	this paper	N/A
reverse primer for *trx-2A(ΔCAT)* AGTCTTGGTCCCAAAGCCTGtgACGGTCCtgACCATT CTGCG	this paper	N/A
reverse primer for *trx-2A* cDNA ccccccACCGGTAGCAGCGAGAACGTCCT	this paper	N/A
forward primer for mito-truck ccccccGCTAGCaaaaATGGAGCCGCGGACAGACG	this paper	N/A
reverse primer for mito-truck ccccccGGTACCCTATCCCCAGAGAAGACTCATGGC	this paper	N/A

forward primer for *sod-2* cDNA ccccccGCTAGCaaaaATGCTTCAAAACACCGTTCG	this paper	N/A
reverse primer for *sod-2* cDNA ccccccGGTACCTTATTGCTGTGCCTTTGCAA	this paper	N/A
reverse primer for *sod-2* cDNA ccccccACCGGTTTATTGCTGTGCCTTTGCAA	this paper	N/A
reverse primer for *trx-2A(ΔMLS)* cDNA CCCCCCGCGATCGCAAAAATGGACATTGATTCTGTTGAAGAT	this paper	N/A
forward primer for *pkc-1A(C524S)* cDNA GGCAGATTTTGGAATGTcCAAGGAAGGAATTAACAAGGA	this paper	N/A
reverse primer for *pkc-1A(C524S)* cDNA TTAATTCCTTCCTTGgACATTCCAAAATCTGCCAAAC	this paper	N/A
forward primer for *sod-2(ΔMLS)* cDNA ccccccGCTAGCaaaaatgACAGGAGTCGCTGCTGTT	this paper	N/A
forward primer for *pkc-1A(C524D)* cDNA GGCAGATTTTGGAATGgaCAAGGAAGGAATTAACAAGGA	this paper	N/A
reverse primer for *pkc-1A(C524D)* cDNA TTAATTCCTTCCTTGtcCATTCCAAAATCTGCCAAAC	this paper	N/A
forward primer for *pkc-1A* cDNA ccccccACCGGTAAATTCTTCAGTAGTCGG	this paper	N/A
reverse primer for *pkc-1A* cDNA ggggggGGTACCTTAGTAGGTAAAATGCGG	this paper	N/A
**Recombinant DNA**		
pJQ04 P*flp-1*::*flp-1(genomic)*	this paper	N/A
pJQ07 P*ges-1*::*egl-3C*::GFP	this paper	N/A
pJQ08 P*flp-1*::*egl-3C*::GFP	this paper	N/A
pJQ33 P*flp-1*::*flp-1(genomic)*::Venus	this paper	N/A
pJQ34 P*flp-1*::*flp-1B*	this paper	N/A
pJQ35 P*flp-1*::*flp-1B(P1+P2+P3+P4)*	this paper	N/A
oJQ37 P*ofm-1*::*npr-4A*	this paper	N/A
pJQ38 P*rab-3*::*npr-4A*	this paper	N/A
pJQ39 P*flp-1*::*flp-1B(P1+P2+P3)*	this paper	N/A
pJQ40 P*flp-1*::*flp-1B(P1+P2)*	this paper	N/A
pJQ41 P*flp-1*::*flp-1B(P1)*	this paper	N/A
pJQ42 P*flp-1*::*flp-1B(SS)*	this paper	N/A
pJQ46 P*flp-1*::mito-miniSOG	this paper	N/A
pJQ47 P*flp-1(513)*::mito-miniSOG	this paper	N/A
pJQ48 P*flp-18*::mito-miniSOG	this paper	N/A
pJQ49 P*flp-1(513)*::*flp-1(genomic)*::Venus	this paper	N/A
pJQ51 P*ttx-3*::*egl-3C*::GFP	this paper	N/A
pJQ52 P*ttx-3*::mito-miniSOG	this paper	N/A
pJQ54 P*ges-1*::*npr-4A*	this paper	N/A
pJQ60 P*ttx-3*::*flp-1(genomic)*::Venus	this paper	N/A
pJQ61 P*ttx-3*::ChR2::GFP	this paper	N/A
pJQ62 P*ttx-3*::*pkc-1A(A160E)*	this paper	N/A
pJQ77 P*ttx-3*::TeTx	this paper	N/A
pJQ79 P*ttx-3*::ss-Venus	this paper	N/A
FP942 pHyPer-dMito vector	Evrogen	cat.# FP942

pJQ107 P*ttx-3*::mito-HyPer	this paper	N/A
pJQ144 P*ttx-3*::*tomm-20*::mCherry	this paper	N/A
pJQ146 P*ttx-3*::*flp-18(genomic)*::Venus	this paper	N/A
pJQ155 P*ttx-3*::*trx-2A*	this paper	N/A
pJQ165 P*ttx-3*::*trx-2A(ΔMLS)*	this paper	N/A
pJQ166 P*ttx-3*::*trx-2A(ΔCAT)*	this paper	N/A
pJQ168 P*ttx-3*::*trx-2A*::GFP	this paper	N/A
pJQ181 P*ttx-3*::mito-truck(*unc-116*::GFP(1-10)::*tomm-7*)	this paper	N/A
pJQ188 P*ttx-3*::*pkc-1A*	this paper	N/A
pJQ190 P*ttx-3*::*sod-2*	this paper	N/A
pJQ191 P*ttx-3*::*sod-2*::GFP	this paper	N/A
pJQ196 P*ttx-3*::*trx-2A(ΔMLS)*::GFP	this paper	N/A
pJQ229 P*ttx-3*::*flp-1(genomic)*::mCherry	this paper	N/A
pJQ236 P*ttx-3*::*pkc-1A(C524S)*	this paper	N/A
pJQ245 P*ttx-3*::*sod-2(ΔMLS)*	this paper	N/A
pJQ249 P*ttx-3*::*sod-2(ΔMLS)*::GFP	this paper	N/A
pJQ270 P*unc-129*::*pkc-1A(C524S)*	this paper	N/A
pJQ271 P*unc-129::pkc-1A*	this paper	N/A
pJQ287 P*ttx-3*::*pkc-1A(C524D)*	this paper	N/A
pJQ293 P*ttx-3*::GFP-linker::*pkc-1A*	this paper	N/A
pJQ296 P*ttx-3*::GFP-linker::*pkc-1A(C524S)*	this paper	N/A
pJQ306 P*ttx-3*::GFP-linker::*pkc-1A(C524D)*	this paper	N/A
